# Mechanisms of structural chromosomal rearrangement formation

**DOI:** 10.1186/s13039-022-00600-6

**Published:** 2022-06-14

**Authors:** Bruna Burssed, Malú Zamariolli, Fernanda Teixeira Bellucco, Maria Isabel Melaragno

**Affiliations:** Genetics Division, Department of Morphology and Genetics, Universidade Federal de São Paulo, São Paulo, SP Brazil

**Keywords:** Structural chromosomal rearrangements, Mechanisms of formation, Non-allelic homologous recombination (NAHR), Non-homologous end-joining (NHEJ), Fork stalling and template switching (FoSTeS), Microhomology-mediated break-induced replication (MMBIR), Chromoanagenesis, Inv dup del, Ring chromosome

## Abstract

Structural chromosomal rearrangements result from different mechanisms of formation, usually related to certain genomic architectural features that may lead to genetic instability. Most of these rearrangements arise from recombination, repair, or replication mechanisms that occur after a double-strand break or the stalling/breakage of a replication fork. Here, we review the mechanisms of formation of structural rearrangements, highlighting their main features and differences. The most important mechanisms of constitutional chromosomal alterations are discussed, including Non-Allelic Homologous Recombination (NAHR), Non-Homologous End-Joining (NHEJ), Fork Stalling and Template Switching (FoSTeS), and Microhomology-Mediated Break-Induced Replication (MMBIR). Their involvement in chromoanagenesis and in the formation of complex chromosomal rearrangements, inverted duplications associated with terminal deletions, and ring chromosomes is also outlined. We reinforce the importance of high-resolution analysis to determine the DNA sequence at, and near, their breakpoints in order to infer the mechanisms of formation of structural rearrangements and to reveal how cells respond to DNA damage and repair broken ends.

## Background

Structural chromosomal rearrangements are abnormalities in the chromosome structure and are known to cause chromosomal and genomic disorders [1–4]. They may affect the phenotype due to abnormal dosage of genes, gene rupture, and gene fusion, among other mechanisms [4–6].

Structural variants result from different mutational mechanisms that include DNA recombination, repair, and replication processes [7]. Studying these mechanisms is crucial as it can help us understand how cells respond to DNA damage and the nature of the DNA sequences involved in these rearrangements. In addition, the study of the rearrangement breakpoints through DNA sequencing allows us to recognize signatures of these processes and identify risk factors for such rearrangements, thus guiding us in inferring their mechanisms of formation [2].

Several methodologies are available to study rearrangements and their breakpoints. G-banding karyotyping allows for the detection of large imbalances of over 5–10 Mb in size [8]. Chromosomal microarray is a technique with much higher resolution [9], which can assess copy number throughout the genome and find submicroscopic alterations [10]. Next generation sequencing (NGS) techniques are used to detect unbalanced and also balanced structural alterations [11, 12]. These techniques are commonly used alongside fluorescent in situ hybridization (FISH), which can unveil the alterations' location and orientation by using fluorescent-labeled DNA probes specific to certain regions of the chromosome [2]. Breakpoint location study can be done using NGS techniques, such as long-read whole genome sequencing [13, 14] and mate-pair sequencing [2, 15], and optical genome mapping technique [16, 17]. For a more comprehensive analysis and to help discover the mechanism of formation of rearrangements, it is vital to use Sanger sequencing, which can confirm breakpoints in the nucleotide level [18–21].

## Recurrent and non-recurrent rearrangements

**Fig. 1:**
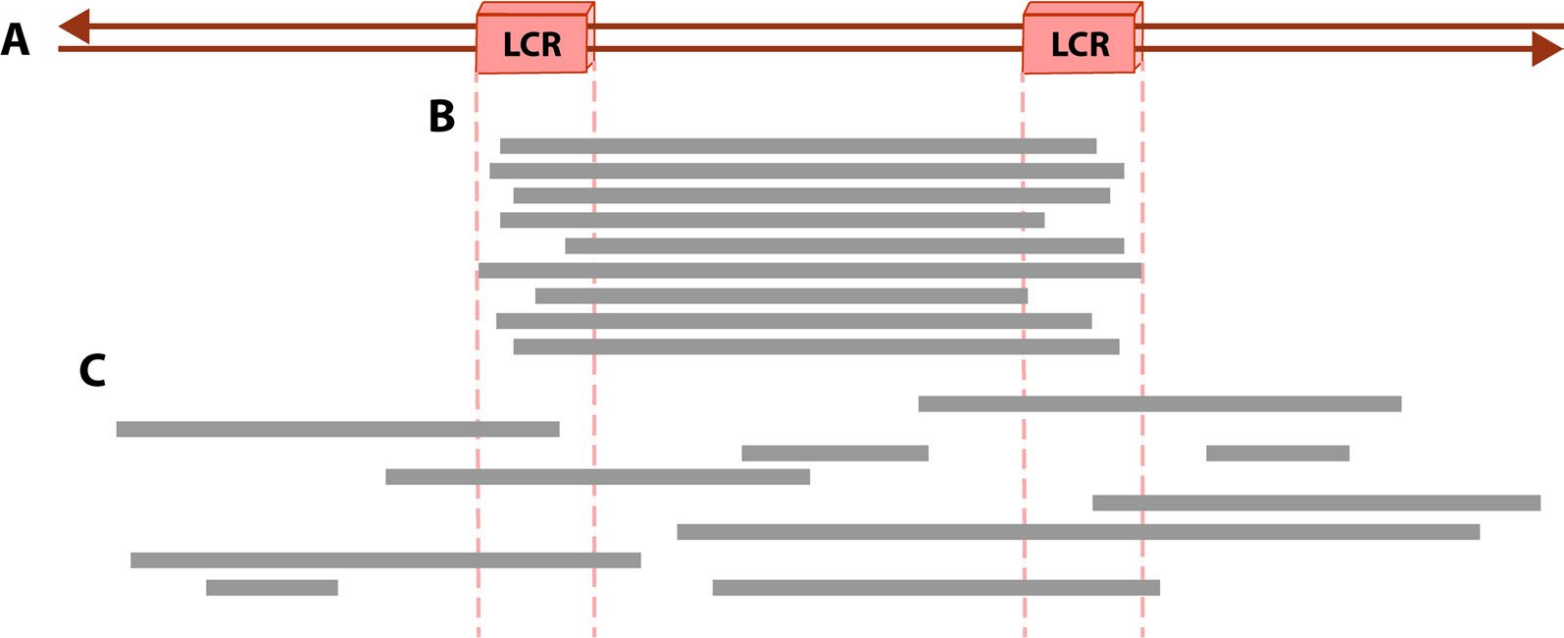
Recurrent and Non-recurrent rearrangements. **A** Representation of a genomic region with two Low Copy Repeats (LCRs). **B** Recurrent rearrangements show similar breakpoints clustered in regions of Low Copy Repeats. **C** Non-recurrent rearrangements show diverse breakpoints and sizes. Each grey bar represents deletions, duplications, or inversions in unrelated individuals

Certain genomic architectural features may lead to genome instability, which can predispose some regions of the genome to rearrange [3, 7]. Recurrent rearrangements (Fig. 1A, B) present similar breakpoints, sizes, and genomic content, which can be shared by unrelated individuals [7, 22]. Non-recurrent rearrangements (Fig. 1C), on the other hand, are unique and each non-related individual has its own breakpoints, size, and genomic content [7]. However, some of them may share a genomic overlap region associated with genomic disorders [6, 7].

Most of these rearrangements arise from recombination or repair mechanisms that occur after a double-strand break (DSB) or replication mechanisms after a replication fork breakage or stalling.

Double strand break is an interruption of the phosphodiester backbone of two complementary DNA strands and can either be two-ended or single-ended [23]. Pathological DSBs may have many causes, which include reactive oxygen species from oxidative metabolism; ionizing radiation, such as X-rays, gamma rays, and UV light; inadvertent action of nuclear enzymes; and when there is physical or mechanical stress on the DNA, such as during the breakage-fusion-bridge cycles after the formation of a chromosome with two centromeres (dicentric chromosome) [6, 24–26]. Pathological DSBs can also be formed when the replication fork encounters a nick, which is a break in a single strand of the DNA backbone [24, 25, 27]. Attempting to correct the DSB, different recombination or repair mechanisms may lead to chromosomal rearrangements.

**Fig. 2:**
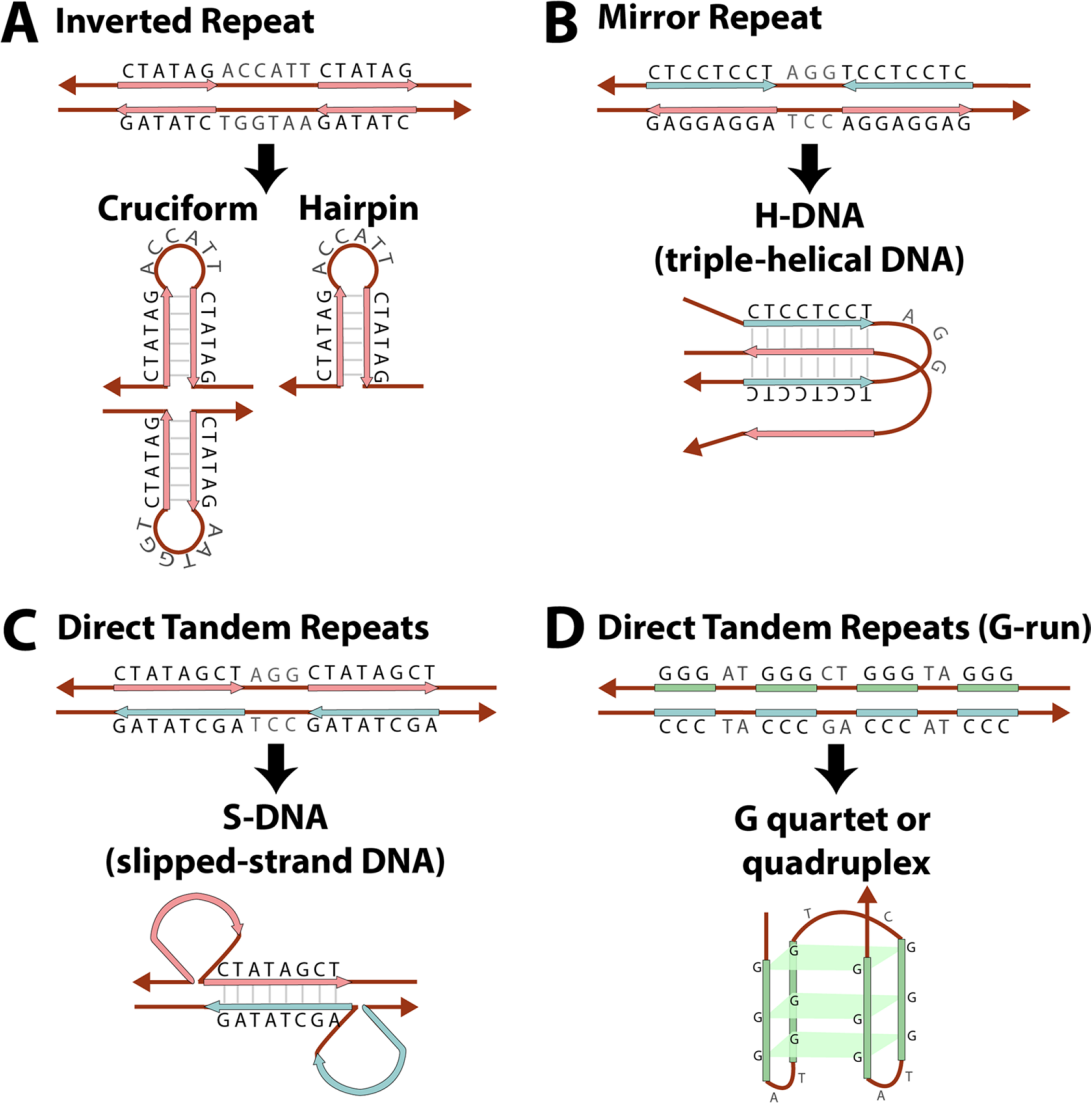
Repetitive sequences and associated non-B DNA structures that can cause replication fork stalling or breakage. **A** Inverted Repeats (equidistant DNA bases are Watson–Crick complements) form cruciform in double-stranded DNA and hairpin in single-stranded DNA, **B** Mirror Repeats (equidistant DNA bases are identical) form H-DNA (triple-helical DNA), **C** Direct Tandem Repeats (simple, noninterrupted repeats) form S-DNA (slipped-stranded DNA), and **D** Direct Tandem Repeats with G-runs form G quartet or quadruplex. Based on [32]

Replication forks can be subjected to breakage or stalling due to replication stress, including DNA lesions, interaction with RNA, and metabolic conditions [28–30]. Alternatively, replication forks can be disturbed by the presence of secondary DNA structures (non-B DNA), usually formed in regions containing repetitive sequences in the genome (Fig. 2) [7, 31]. For example, inverted repeats have been associated with the formation of cruciform figures in double-stranded DNA or hairpins in single-stranded DNA; mirror repeats can form H-DNA (triple-helical DNA); and, depending on their base composition, direct tandem repeats can form quadruplex, left-handed Z-DNA, and slipped-strand DNA (S-DNA) [7, 31, 32]. These secondary DNA structures have been implicated with inhibition of a variety of DNA polymerases and confusion of the DNA replication machinery, which causes replication forks to stall or break and allows for template-switching events that can create complex rearrangements [29, 31]. Sometimes, they occur due to the presence of microhomology, which are short DNA segments with homology in their sequences, commonly defined as a series of nucleotides (< 70) near both broken ends involved in the rearrangement [26].

Here, we review the mechanisms of structural rearrangement formation and highlight the importance of using high-resolution analysis to find and sequence their breakpoints.

## Mechanisms of structural chromosomal rearrangement formation

### Non-allelic homologous recombination (NAHR)

Most recurrent rearrangements are caused by a mechanism named Non-Allelic Homologous Recombination (NAHR) that occurs between Low Copy Repeats (LCRs), thus causing breakpoint clustering near these regions [3, 7]. Low Copy Repeats, also known as Segmental Duplications, are DNA blocks of 10 to 400 kb in size with over 97% identity between sequences [4, 33]. They can contain genes, pseudogenes, repeat gene clusters, or other paralogous sequences [3, 33].

**Fig. 3:**
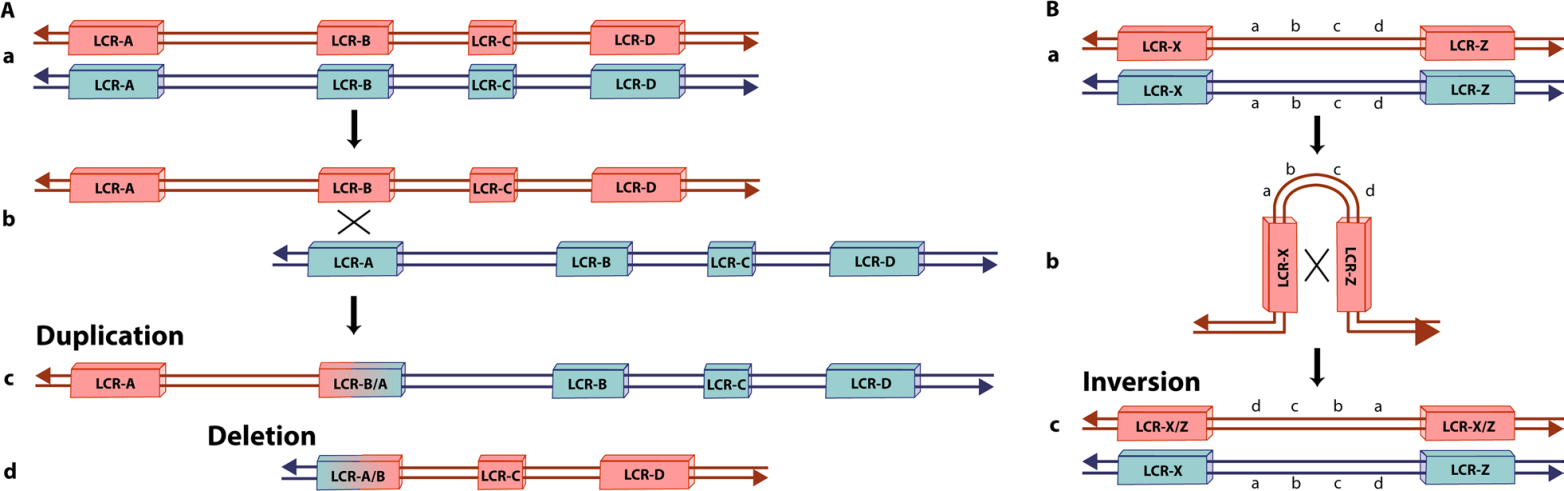
Non-Allelic Homologous Recombination (NAHR) mechanism. **A** NAHR leading to the formation of duplication and deletion: (a) Normal chromosome pairing and alignment of Low Copy Repeats (LCRs) in the same orientation. (b) A misalignment between LCRs due to their high level of sequence identity leads to an unequal crossing over event that can generate (c) a duplication and (d) a deletion. The scheme represents the LCRs of region 22q11.2. NAHR between LCR-A and LCR-B leads to a deletion seen in 8% of the cases of 22q11.2 deletion syndrome. **B** NAHR leading to the formation of inversions: (a) Normal chromosome pairing and alignment of Low Copy Repeats (LCRs). LCR-X and LCR-Z present similar DNA sequences but in opposite orientations. (b) A misalignment between LCRs due to their high level of sequence identity leads to an unequal crossing over event that can generate (c) an inversion

LCRs can act as substrates for homologous recombination due to their sequence similarities. Their size, degree of homology, orientation, and relative arrangement affect genome architecture resulting in unstable regions, which become prone to rearrangements [33]. NAHR (Fig. 3) can occur after a DSB during meiosis or mitosis when non-allelic copies of LCRs erroneously align due to their high level of sequence identity. This misalignment causes an unequal crossing over event generating genomic rearrangement in the daughter cells [3, 6].

NAHR is a mechanism that can occur intrachromosomal, intrachromatid or interchromosomal [33]. NAHR between LCRs in direct orientation in sister chromatids of the same chromosome (intrachromosomal or interchromatid) causes reciprocal duplications and deletions (Fig. 3A), whereas inversions are formed when LCRs are found in the opposite orientation (Fig. 3B). The same happens for NAHR between LCRs in the same chromatid (intrachromatid) but no duplications are formed [6]. NAHR between LCRs present in different chromosomes (interchromosomal) causes translocations.

LCRs have been identified in several chromosomes and are associated with several genomic diseases due to copy number variations (CNVs) formed through NAHR [34]. For example, NAHR between LCRs located in chromosome 22q (named from A to D) causes the 22q11.2 deletion syndrome, also known as DiGeorge Syndrome [35]. The most common deletion of 3 Mb (90% of affected patients) results from NAHR occurring between LCR22-A and LCR22-D, the LCRs with the most significant identity, while a smaller deletion of 1.5 Mb (8% of patients) involves LCR22-A and LCR22-B (Fig. 3A). Other rearrangements caused by NAHR between LCRs include the 7q11.23 deletion in Williams–Beuren syndrome, 15q11.2 deletion in Prader–Willi syndrome and Angelman syndrome, and 17p11.2 deletion in Charcot–Marie–Tooth disease type A [33].

Mobile elements, which correspond to 45% of the human genome [36, 37], have also been implicated in the formation of non-recurrent rearrangements through NAHR. They include, among others, the short interspersed element (SINE) Alu, and the long interspersed element-1 LINE-1 (or L1). Alu elements are around 300 bp long and constitute 10% of the genome, whereas LINE-1 elements are around 6 kb in size and constitute 20% of the genome. These elements' amplification happens through retrotransposition with LINE-1 elements encoding proteins needed for their mobility (autonomous retrotransposons) and for the mobility of non-autonomous retrotransposons such as Alus [36–38].

Startek et al. [39] analyzed the involvement of LINE-1 elements in NAHR. They found that 96% of identity is needed between their homologous sequences to mediate NAHR and that 1 kb of homology seems to be enough to facilitate the occurrence of this mechanism. Furthermore, since mobile elements are abundant in the genome, they highlight that a large part of the genome (82% in their analysis) is susceptible to rearranging through NAHR.

Non-recurrent rearrangements formed through NAHR between Alu elements have been reported, for example, in familial hypercholesterolemia [40], subtelomeric interstitial deletions [41], and Smith-Magenis syndrome [42]. In addition, NAHR between LINE-1 elements has been described, for example, in phosphorylase kinase deficiency [43], Mesomelia-synostoses syndrome [44], and translocations [45].

However, it is likely that the number of rearrangements mediated by NAHR using Alu and LINE-1 elements is underestimated due to the techniques used to characterize the rearrangements [39, 41]. They are often missed when patients' DNA is submitted only to array analysis and not to sequencing.

### Non-homologous end-joining (NHEJ)

**Fig. 4:**
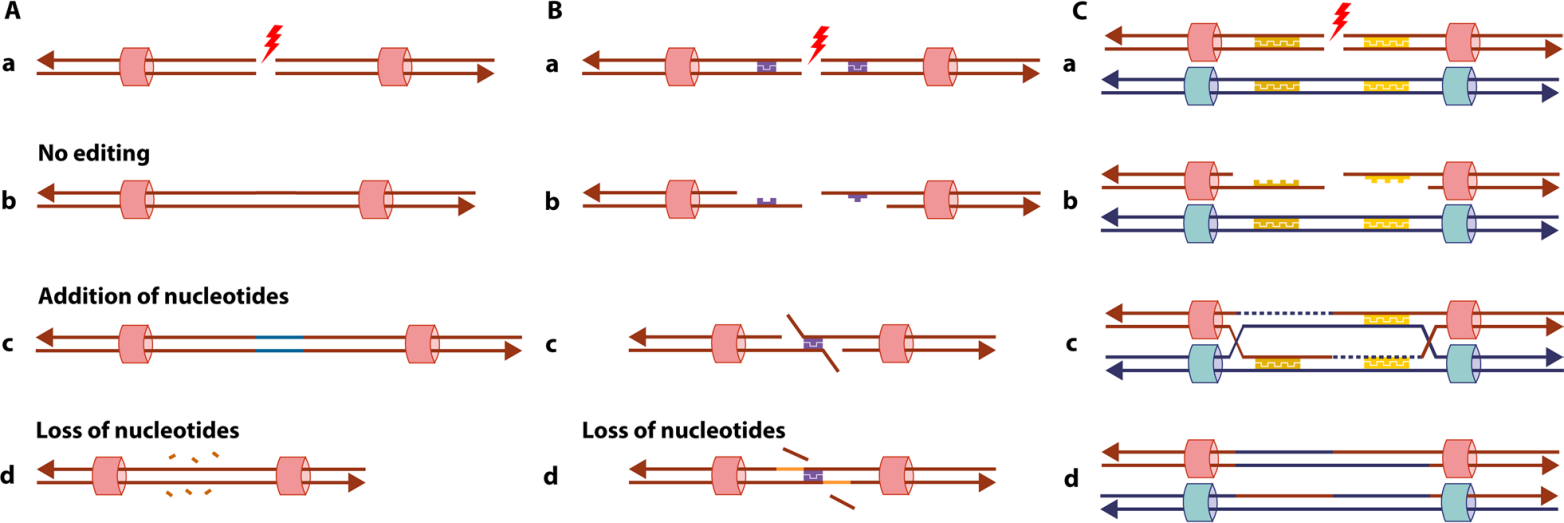
Mechanisms of double-strand break (DSB) repair. **A** Canonical Non-Homologous End-Joining (c-NHEJ) mechanism: The repair of (a) the DSB is done by molecularly bridging and rejoining the broken ends. This can happen (b) without nucleotide edition or through the editing of the broken ends with (c) addition or (d) loss of nucleotides. **B** Microhomology-Mediated End-Joining (MMEJ) mechanism: After (a) the DSB, (b) a 5′ to 3′ resection results in two 3′ single-stranded overhangs with exposed nucleotides of microhomology (purple), which (c) anneal. (d) After the trimming of the 3′ strands, the gaps are filled (light orange) and ligated, resulting in the repair with a deletion. **C** Homologous Recombination (HR) mechanism: after (a) a DSB in one of the sister chromatids, (b) a 5′ to 3′ resection creates a 3′ overhang with an exposed region of homology (yellow), (c) which helps the broken end to invade its sister chromatid and anneal, allowing for the start of DNA synthesis. (d) The DSB can be solved using homologous chromosomes without errors, therefore without the creation of rearrangements. It is important to note that HR can lead to rearrangements when it uses repetitive elements and not the homology located at the sister chromatid

The main pathway for repairing of two-ended double-strand breaks is the Non-Homologous End-Joining (NHEJ) mechanism [24, 46]. The canonical NHEJ (c-NHEJ) mechanism (Fig. 4A) can function throughout the cell cycle and does not require any homology at the breakpoints. After detecting the DSB, both broken DNA ends are molecularly bridged, modified, and then rejoined [6]. C-NHEJ is known for being imprecise, which means that information scars (gain or loss of nucleotides) may be seen in the junction points [6, 7, 24, 26, 28, 46]. To facilitate ligation of the broken ends, nucleotides can be added to mimic microhomology since most causes of DSB form blunt ends that do not share any. To the same end, nucleotides can also be lost with little additional consequences since the loss of nucleotides is sometimes a consequence of some causes of DSB, such as ionizing radiation [24]. This mechanism is responsible for forming different rearrangements such as deletions, duplications, inversions, and translocations [6, 7, 26, 28].

An alternative form of end-joining (alt-EJ or a-NHEJ) mechanism, known as Microhomology-Mediated End-Joining (MMEJ), has also been described to repair two-ended DSBs [24, 26, 46]. MMEJ relies on the presence of microhomology between the broken ends to perform the repair (Fig. 4B). This mechanism starts with the resection of both 5′ ends involved in the break to obtain two 3′ single-stranded overhangs. As a result, stretches of microhomology are exposed on both ends, helping them anneal and ligate. The resulting gaps due to the 5′ resection are filled and DNA becomes double-stranded again. The region around the original break is deleted, therefore this mechanism is error-prone and can also form rearrangements [26].

It has also been reported that c-NHEJ can use microhomology to rejoin the broken ends. However, as explained previously, it can function without it, whereas MMEJ only functions in the presence of microhomology [25, 26]. MMEJ is believed to function throughout the cell cycle as NHEJ also does, but the proteins needed for each mechanism are different and their availability directs the cells as to which one should be performed to repair their DSBs [24–26, 46]. Since only the final result can be seen when sequencing the breakpoint, it is not always possible to pinpoint the exact mechanism of formation when comparing c-NHEJ and MMEJ.

NHEJ is considered the major mechanism responsible for the formation of balanced chromosomal alterations [20, 47]. Moysés-Oliveira et al. [20] reported mutational signatures of c-NHEJ in the breakpoints of six out of ten patients in a study of X-autosome balanced translocations. NHEJ also has the potential to form different types of rearrangements, such as inverted duplications associated with terminal deletions, ring chromosomes, and chromoanagenesis, which are described in the posterior sections of this review.

Repetitive sequences such as Alu, LINEs, LCRs, and palindromic repeats have been associated with non-recurrent deletions formed through NHEJ [6, 42]. An example happens in the dystrophin gene located at Xq21, a region with a high recombination rate and a high repetitive sequence percentage [48]. Nobile et al. [49] and Toffolatti et al. [48] studied patients with Duchenne and Becker muscular dystrophies caused by deletions in the gene and found that 42% of their breakpoints mapped within such repetitive elements with short insertions, duplications, and microhomology at the junctions pointing to NHEJ as the mechanism of formation. LCRs may predispose to DSBs, which facilitates not only NAHR but also NHEJ [42]. Inoue et al. [50] studied three deletions in the *PLP1* gene, located at Xq22, and found that the distal breakpoints of two of them fell within LCRs and NHEJ was the proposed mechanism of formation due to the insertion of nucleotides at the junction points. The third one was formed through NAHR between Alu elements and led to the formation of a translocation t(X;9). The same region of chromosome 22 involved with the 22q11.2 deletion syndrome also presents palindromic AT-rich repeats, which facilitates interaction with other AT-rich repeats on chromosome 11 to form the recurrent translocation t(11;22) through NHEJ resulting in Emanuel syndrome [51–53].

It is relevant to note that not all DSBs lead to chromosomal rearrangements since they can be healed by Homologous Recombination (HR). This repair mechanism is often error-free and requires DNA homologous sequences, which are identical and span around 300 bp, located in the same position either in the sister chromatid or the homologous chromosome to repair the DSB (Fig. 4C) [26, 54]. However, chromosomal rearrangements can occur when the homologous sequence is found in different chromosomal positions or when repetitive sequences are used [54].

### Replication-based mechanisms

Replication-based mechanisms have been proposed to explain complex rearrangements containing multiple breakpoints, insertions of DNA segments, and microhomology at the breakpoint junctions [7, 26]. They arise due to a change of single-stranded DNA template during replication, which is known as template switching, after replication fork stalling or breakage.

**Fig. 5:**
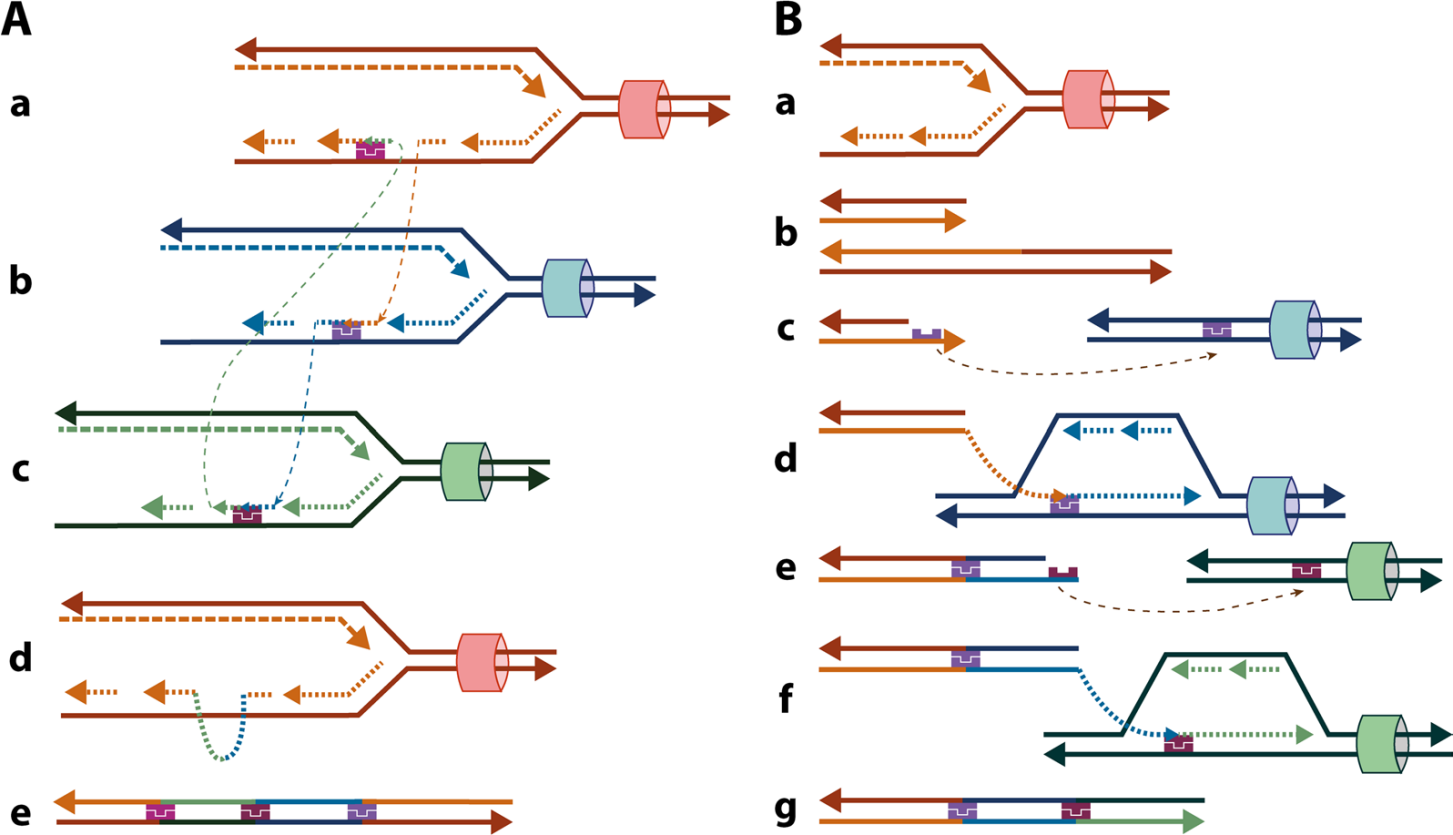
Replication mechanisms. **A** Fork Stalling and Template Switching (FoSTeS) mechanism: (a) When a replication fork stalls, (b) the lagging strand disengages from its original template and, due to the presence of microhomology (purple), invades and switches to another template (dashed line) at another active replication fork and restarts DNA synthesis. (c) The nascent lagging strand can disengage again and invade other replication forks. Eventually, the strand can return to its original template and (a,d) restart synthesis. (e) The final product contains segments from different parts of the genome that were brought together due to microhomology. **B** Microhomology-Mediated Break-Induced Replication (MMBIR) mechanism: (a) A replication fork collapses when it encounters a DNA lesion, forming (b) a single-ended double-strand break. (c) A 5′–3′ resection creates a 3′ overhang with an exposed region of microhomology (purple), which serves as a template for (d) the invasion of a different region of the genome, where DNA synthesis is restarted. (e, f) The process can be repeated, and other regions of the genome can be invaded due to the presence of microhomology. (g) The final product presents a complex rearrangement with distinct parts of the genome united due to microhomology. In FoSTeS and MMBIR, the low processivity of the DNA polymerase leads to constant strand switching, especially at the beginning of the process, which may lead to short insertions in the junction points. As the invasion goes on, the DNA polymerase is switched and becomes more processive, allowing the replication to proceed until the end of the chromosome. The Break-Induced Replication (BIR) mechanism process is similar to MMBIR but uses larger homologous sequences instead of microhomologies

Replication fork stalling can lead to the mechanism named Fork Stalling and Template Switching (FoSTeS) (Fig. 5A). During replication, the replication fork pauses at one position. The lagging strand disengages from the original template and, due to the presence of microhomology, switches to another template at another active replication fork and restarts DNA synthesis. In the new fork, DNA is copied, and the nascent strand can disengage again and invade other replication forks and/or return to its original one, all based on the presence of microhomology, which allows for this transfer and annealing of the strand to occur many times leading to numerous invasions. Switching to a replication fork located downstream, known as forward invasion, results in deletion, whereas switching to one located upstream, known as backward invasion, results in duplication. Switching to a different chromosome leads to translocations. Inverted segments can also be formed since the lagging strand can invade a replication fork advancing in either direction (5′ to 3′ or 3′ to 5′) [6, 29].

Replication fork breakage or collapse can lead to two mechanisms: Break-Induced Replication (BIR) and Microhomology-Mediated Break-Induced Replication (MMBIR) (Fig. 5B). During replication, the replication fork breaks, and a single-ended DSB is formed. Resection of the 5′ end generates a 3′ single-stranded overhang that can invade another genomic region. This invasion requires long stretches of homologous DNA sequences for BIR and microhomology for MMBIR in the invaded segment. A replication fork is established in this new region and DNA synthesis continues. As with FoSTeS, multiple dissociations and invasions can take place until the end of a chromosome is reached. It is interesting to note that previously described mechanisms, such as NHEJ and MMEJ, cannot fix the problem of the single-ended DSB since there is no second end that could be ligated to it. As a result, the free 3′ keeps invading regions of the genome as if looking for its other side [30]. Switching to an upstream position leads to duplication, to a downstream position leads to deletion, to a sequence in the opposite orientation leads to inversions, and to a different chromosome leads to translocations [26, 30]. Besides the amount of homology needed for the invasion, another element that sets BIR and MMBIR apart is the proteins needed for each mechanism to occur [30].

Due to the constant dissociations and strand switching, complex chromosomal rearrangements can be formed via these three replication-based mechanisms. This happens due to the low processivity of DNA polymerases at the beginning of the replication process [30], meaning that the enzyme cannot remain attached to DNA for long periods and ends up releasing the strands. As a result, 35% of the breakpoint junctions, inferred to be formed by these mechanisms, contain short inserted sequences (< 100 bp) [7, 55]. After a few cycles of invasion, the replication fork becomes more processive due to the switch of DNA polymerases involved and the replication can continue until the chromosome end [30]. Also, strand switching can happen to regions that are physically close but separated by large linear distances. Complex genome architecture such as LCRs and repetitive sequences (which can form non-B DNA structures) are able to bring these distant regions together due to their sequence similarities. This allows for the formation of complex rearrangements that involve distinctive regions of the genome [29]. The location of these structures frequently matches the location of a template switch or a breakpoint, which could mean that the occurrence of non-recurrent rearrangements is not always random [7, 56].

Known diseases caused by rearrangements formed through replication mechanisms have been reported. For example, Carvalho et al. [55] found that nine out of 31 individuals with the *MECP2* Duplication Syndrome had complex rearrangements with direct and inverted insertions at the breakpoint formed concomitantly with the duplication. In this case, a replication-based mechanism was suggested, meaning that the DNA polymerase slippage happened within a replication fork where DNA synthesis occurred in both leading- and lagging-strands. The authors implicate BIR in their formation.

### Chromoanagenesis

Chromoanagenesis, meaning chromosome rebirth (from Greek), is a term coined by Holland and Cleveland [57] and used to encapsulate three distinct mechanisms (Chromothripsis, Chromoanasynthesis, and Chromoplexy). They constitute extremely complex and massive chromosomal alterations where a single catastrophic event is responsible for multiple structural rearrangements in one or more chromosomes [28, 57–59].

**Fig. 6:**
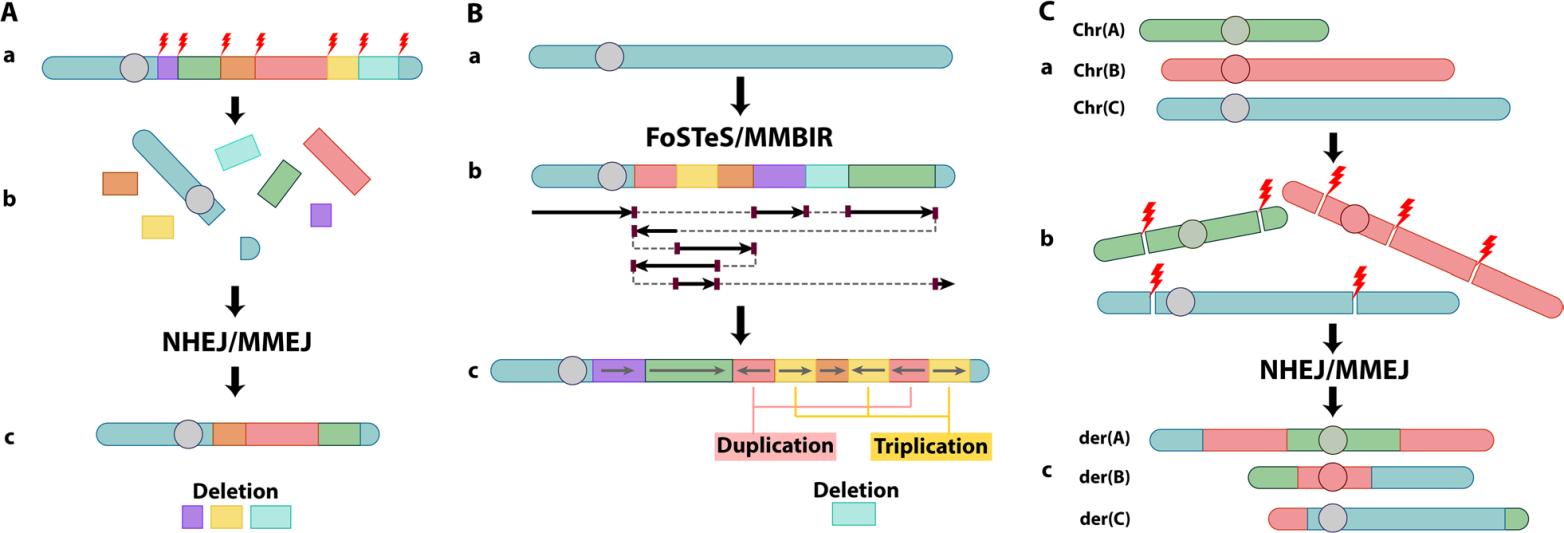
Chromoanagenesis. **A** Chromothripsis: (a) A missegregated chromosome is encapsulated inside a micronucleus and suffers multiple double-strand breaks, leading to (b) the shattering of the chromosome. (c) The chromosome is reassembled by c-NHEJ or MMEJ and reincorporated in the nucleus. Deletions due to loss of DNA fragments can occur. **B** Chromoanasynthesis: (a) A chromosome undergoes (b) DNA segment re-synthesis in a process that involves FoSTeS or MMBIR, forming (c) a new chromosome, which can present inversions, deletions, duplications, and triplications. **C** Chromoplexy: (a) Different chromosomes suffer (b) double-strand breaks and, after recombination by NHEJ or MMEJ, (c) form chromosomes with translocations. Based on (59)

The first mechanism is Chromothripsis (Fig. 6A), meaning chromosome shattering. It was first described in cancer cells [60] and usually involves one chromosome, wholly or only one arm [28, 59, 61]. The formation of multiple clustered DSBs leads to local shattering, causing the chromosome to break into small fragments which are then randomly reassembled in a different order and orientation when compared to the original chromosome [28, 59, 61–63]. The whole process may happen inside a micronucleus, where missegregated chromosomes are encapsulated, pulverized, and then reassembled by c-NHEJ or MMEJ with the complex derivative chromosome reincorporated in the main nucleus [28, 59, 61, 64]. Deletions can be formed since some fragments may be lost if they are not incorporated in the process, but duplications are not found [28, 61–63]. Distinguishable characteristics of chromothripsis include: (i) clustering of breakpoints showing signs of c-NHEJ or MMEJ, (ii) low DNA copy number changes with oscillation of copy number pattern between one (deleted fragments) and two copies, (iii) only one haplotype affected, and (iv) preservation of heterozygosity [59, 61, 62, 65].

The second is Chromoanasynthesis (chromosome reconstitution) (Fig. 6B), a mechanism that involves a single chromosome or a chromosome locus [2, 59, 62, 66]. The chromosome undergoes DNA segment re-synthesis in a process that involves FoSTeS or MMBIR to form a new rearranged chromosome [59, 62]. Unlike chromothripsis, it results in copy number gain, with complex rearrangements involving duplications and triplications, sometimes combined, and does not require micronucleus formation [62]. Distinguishable characteristics of chromoanasynthesis include: (i) the formation of localized multiple copy-number changes, including deletions, duplications, triplications, inversions, and translocations, and (ii) microhomology at the breakpoints, which can be many and formed in a single event [58, 59, 62].

The third mechanism is Chromoplexy (Fig. 6C), meaning chromosome restructuring. It was first described in prostate cancer [62, 67] and involves more than two chromosomes [59, 62]. The process relies on a chain of rearrangements involving breaking and rejoining through c-NHEJ or MMEJ of DNA segments from different chromosomes, thus leading to the formation of translocations [59, 62]. The rearrangements are usually copy number neutral. However, they may present minimal DNA gain or loss at the junction points [59, 62]. Distinguishable characteristics of chromoplexy include: (i) the involvement of multiple chromosomes, (ii) the creation of fewer breakpoints in one chromosome compared to chromothripsis, and (iii) the formation of translocations with no significant copy number alterations [28, 59, 62].

Mimicking the chromoanagenesis name, Iourov et al. [68] coined the term Chromohelkosis (chromosome ulceration), which is also a mechanism that creates chromosome rearrangements and chromosomal instability. The process starts with a regular imbalance (CNV), which initiates genomic instability at adjacent loci and, due to defective DNA repair mechanisms, forms mosaic imbalances by a "wreckage" of both of its breakpoints. This mechanism can explain the authors' findings of concomitant regular and mosaic chromosome imbalances located at the same locus.

### Mechanisms involved in the formation of inverted duplication/terminal deletion (inv dup del) rearrangements

The development of molecular cytogenetic methods allowed for a better characterization of rearrangements. Several alterations previously classified as simple terminal deletions were actually inverted duplication associated with a terminal deletion, a rearrangement commonly known as inv dup del. Those inv dup del rearrangements were first described by Weleber et al. [69] and have since been identified for most chromosomes [70–73]. In some cases, a normal copy number region (disomic spacer) can be found between the non-inverted and inverted segments [70–73].

Four mechanisms have been proposed to explain the formation of such rearrangements. All of them rely on the formation of a dicentric chromosome, which is later broken to form a duplicated and deleted chromosome. The difference between these mechanisms is in the events that lead to the formation of the dicentric [70] and in the presence or absence of the disomic spacer between the two copies of the duplicated segment [70, 71].

**Fig. 7:**
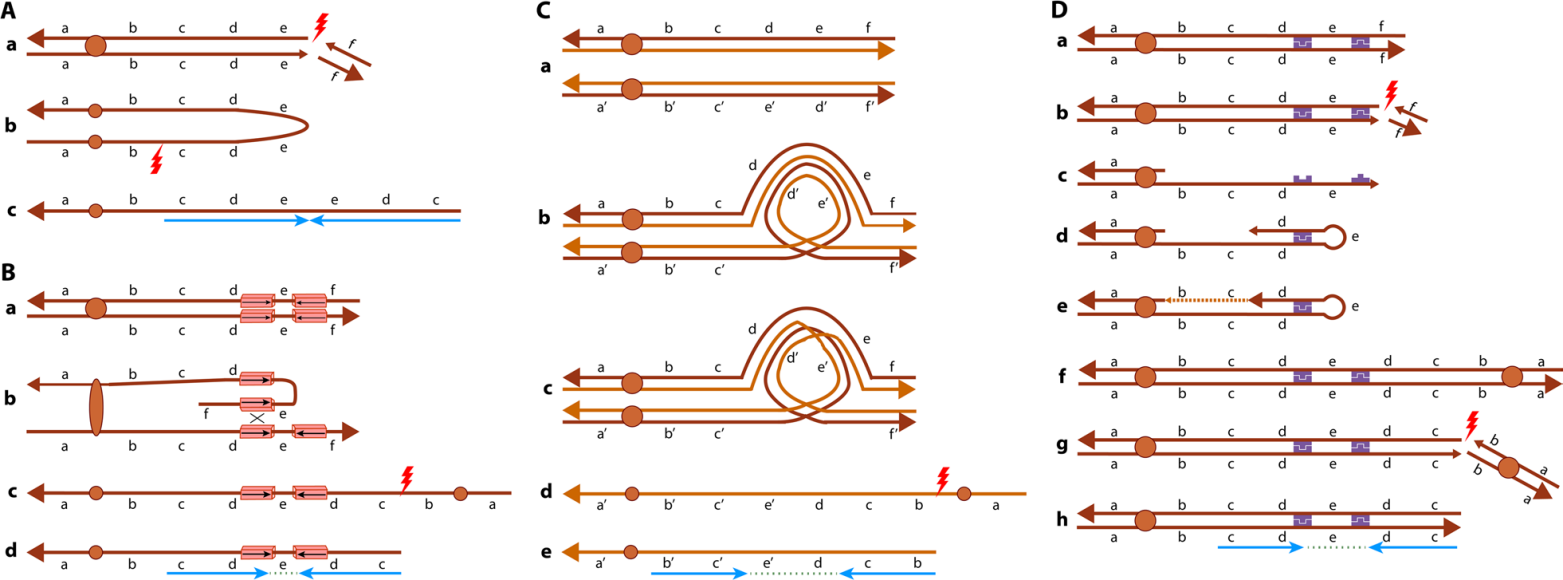
Mechanisms of formation of inverted duplications associated with terminal deletions (inv dup del rearrangements). **A** U-type Exchange Mechanism: (a) a double-strand break in sister chromatids leads to a terminal deletion. (b) Fusion of the broken ends forms a symmetric U-type structure and produces a dicentric chromosome, which undergoes another double-strand break forming the (c) inv dup del chromosome without the spacer between the duplicated region (shown as blue arrows). **B** Low Copy Repeat-dependant Mechanism: (a) LCRs in inverted orientation located in sister chromatids lead to (b) a partial folding of a chromatid onto itself so that the LCRs can pair and align with the other chromatid. A crossing over event can happen between them and form (c) a dicentric chromosome, which undergoes a double-strand break and forms the (d) inv dup del chromosome, which presents a spacer flanked by the LCRs between the duplicated region (shown as blue arrows). **C** Paracentric Inversion-dependant Mechanism: (a) Two homologous chromosomes with one (bottom) presenting an inversion (e′–d′). (b) Formation of an inversion loop to allow for proper chromosome pairing with the normal homolog during meiosis. (c) A crossing over event happens within the loop and leads to the formation of (d) a dicentric chromosome, which undergoes a double-strand break and forms the (d) inv dup del chromosome, which presents a spacer between the duplications (shown as blue arrows). **D** Fold-back Mechanism: (a) Chromosome with microhomologies (shown in purple). (b) A double-strand break forms the terminal deletion. (c) A 5′ to 3′ resection creates a 3′ overhang with exposed microhomologies. (d) Due to the microhomologies, the 3′ overhang folds back onto itself and allows for intrastrand pairing. (e) DNA synthesis fills the resected gap forming a monocentric fold back chromosome, and (f) DNA replication forms a dicentric chromosome, which suffers a (g) double strand break forming an (h) inv dup del chromosome, which presents a spacer between the duplicated region (shown as blue arrows)

In the first mechanism, known as U-type exchange (Fig. 7A), a double-strand break in two sister chromatids is repaired through NHEJ, which leads to the fusion of the broken ends forming a symmetric U-type structure, thus producing the dicentric chromosome [70–72, 74]. It has also been reported that replication mechanisms, such as FoSTeS or MMBIR, can be responsible for the U-type exchange formation due to the presence of microhomology between sister chromatids [15]. A critical aspect of this mechanism is that there is no spacer between the two copies of the duplicated segments [70, 71].

The second mechanism is dependant on Low Copy Repeats, which makes the region prone to NAHR (Fig. 7B). LCRs of inverted orientation in the same chromosome arm lead to a partial folding of the homologous chromosome onto itself so that the LCRs can pair and align. A crossing over event can happen between them and form the dicentric chromosome. In this mechanism, a disomic spacer flanked by the LCRs can be found between the inverted and non-inverted segments [70, 73, 75].

The third mechanism requires the presence of a paracentric inversion, derived from two breaks in the same chromosome arm, and the inversion of a segment not involving the centromere [76], which is present in one of the parents (Fig. 7C) [70, 77]. During meiosis, the formation of an inversion loop allows the paracentric inverted chromosome to pair with its normal homolog. A crossing over event may happen within the loop, creating the dicentric chromosome. In this mechanism, a disomic spacer can be found between the duplicated segments [70].

The fourth, and most recently described mechanism by Hermetz et al. [71], was named Fold-back (Fig. 7D). In this mechanism, an initial double-strand break causes a terminal deletion. Since this region is left unprotected without a telomere, the DNA from the free end can suffer a 5′–3′ resection creating a 3′ overhang that can fold back and intrastrand pair with itself in a region of microhomology. DNA synthesis can fill the resected gap, thus forming a monocentric fold-back chromosome. Following DNA replication, the dicentric chromosome is created and it presents a disomic spacer between the two copies of the duplication which corresponds to the fold-back loop region. The size of the spacer depends on the amount of DNA resected and the distance between the microhomologies, whose amount needed for intrastrand pairing is unknown. Hermetz et al. [71] also highlighted that the inverted duplication and the terminal deletion are formed in a single event and constitute an intrachromosomal rearrangement that involves intrastrand pairing within a sister chromatid.

Through one of these four mechanisms, a dicentric chromosome is formed. This dicentric possesses two centromeres, therefore it is unstable during cell division. During anaphase, the dicentric is pulled to opposite poles of the cell and breaks, resulting in two monocentric reciprocal products: one with a terminal deletion and one with the inverted duplication followed by a terminal deletion [70, 71, 74, 78]. It is important to note that the size of the duplication depends on where the break occurs between the two centromeres of the dicentric chromosome [73].

The resulting chromosomes are left with no telomeres, leaving them vulnerable due to their genomic instability. However, there are ways in which the cell can repair and stabilize the chromosomes [15, 70–73, 78–82]. The first way is by adding a new telomere via the telomerase action, which would create a simple inverted duplication followed by a terminal deletion. The second way is through telomere capture, in which the chromosome can acquire a new telomere sequence from its sister chromatid or a non-homologous chromosome end, thus inducing the formation of a translocation. The third way is through circularization, forming a ring chromosome due to fusion with the other arm of the chromosome. Milosevic et al. [72] also described break-induced replication as a mechanism that can stabilize broken chromosome ends.

The second and third mechanisms can cause the most recurrent inv dup del rearrangement: the inv dup del(8p). The 8p region presents the two olfactory receptor gene clusters, known as REPeat Proximal (REPP) and REPeat Distal (REPD), which flank a 5 Mb segment of 8p23.1 and leaves this region vulnerable to genomic rearrangements [75]. LCRs are found within REPP and REPD and facilitate NAHR to form inv dup del. As a result, all rearrangements formed through this mechanism have similar size deletions (7–8 Mb, the distance between 8pter and REPD) and disomic spacers (4–5 Mb, the distance between REPD and REPP), which are flanked by the repeats [70, 71, 73, 75, 82]. A polymorphic paracentric inversion of 8p23.1 between REPD and REPP, usually present in the mother, promotes the occurrence of the third mechanism to form inv dup del(8p) in the offspring [15, 70, 75, 83].

Kato et al. [15] analyzed ten cases of inv dup del in nine different chromosome regions. Two of them had the recurrent inv dup del(8p) formed through NAHR with similar breakpoints and the large disomic spacer (5 Mb) between the duplications. The remaining eight cases featured smaller disomic spacers ranging from 1 to 5 kb, which appears to be the most common size of this region when other studies were also compared [15, 70, 71].

### Mechanisms involved in ring chromosome formation

**Fig. 8:**
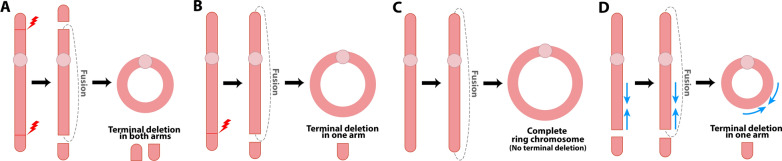
Ring Chromosome Formation. **A** Two terminal double-strand breaks in each chromosome arm and subsequent fusion of the broken ends lead to the formation of a ring chromosome with terminal deletions in both arms. **B** A terminal double-strand break in one arm and subsequent fusion of the broken end with the opposite arm's telomeric or subtelomeric region leads to the formation of a ring chromosome with terminal deletions in one arm. **C** Fusion of the telomeric or subtelomeric regions of a chromosome without terminal deletions leads to the formation of a complete ring chromosome. **D** An inv dup del chromosome can be stabilized via circularization after fusion of the end of the inverted duplication with the opposite arm's telomeric or subtelomeric region, leading to the formation of a ring chromosome with inverted duplication (blue arrows) and terminal deletion

Ring chromosomes are rare chromosomal alterations formed by circular DNA molecules described for all human chromosomes [80, 84, 85] and can be formed through a few described mechanisms (Fig. 8).

The classic mechanism of formation involves two terminal double-strand breaks, one in each chromosome arm, and subsequent fusion of the broken ends. Different types of rings are originated depending on the site where the breaks occur, that is, along the chromosome arms, or at the telomeric or subtelomeric regions: ring chromosomes with terminal deletions in both arms; rings with a terminal deletion in only one chromosome arm [80, 86–88]; or complete ring chromosomes without any loss of significant genetic material [80, 86–88].

As mentioned in the previous section, circularization is a way to stabilize chromosomes with terminal deletions [70, 71, 78, 79]. As a result, ring chromosomes can also be formed through any of the four mechanisms described for inv dup del formation followed by circularization of the chromosome involved. In this case, the ring will have an inverted duplication associated with a terminal deletion [80, 86, 87]. Chai et al. [89] reported a patient with a complex inv dup del ring chromosome 9 formed due to the presence of inverted repeats and microhomologies, which allowed for intrastrand fold-back and DNA synthesis.

More complex ring chromosomes, such as dicentric rings, interlocked rings, and other structural conformations, can be formed due to sister chromatid exchanges during mitosis [80]. Thus, due to their genetic instability, rings can be subjected to secondary genetic material gain or loss [80, 87].

## Breakpoint analysis and the inference of the mechanism of rearrangement formation

**Fig. 9:**
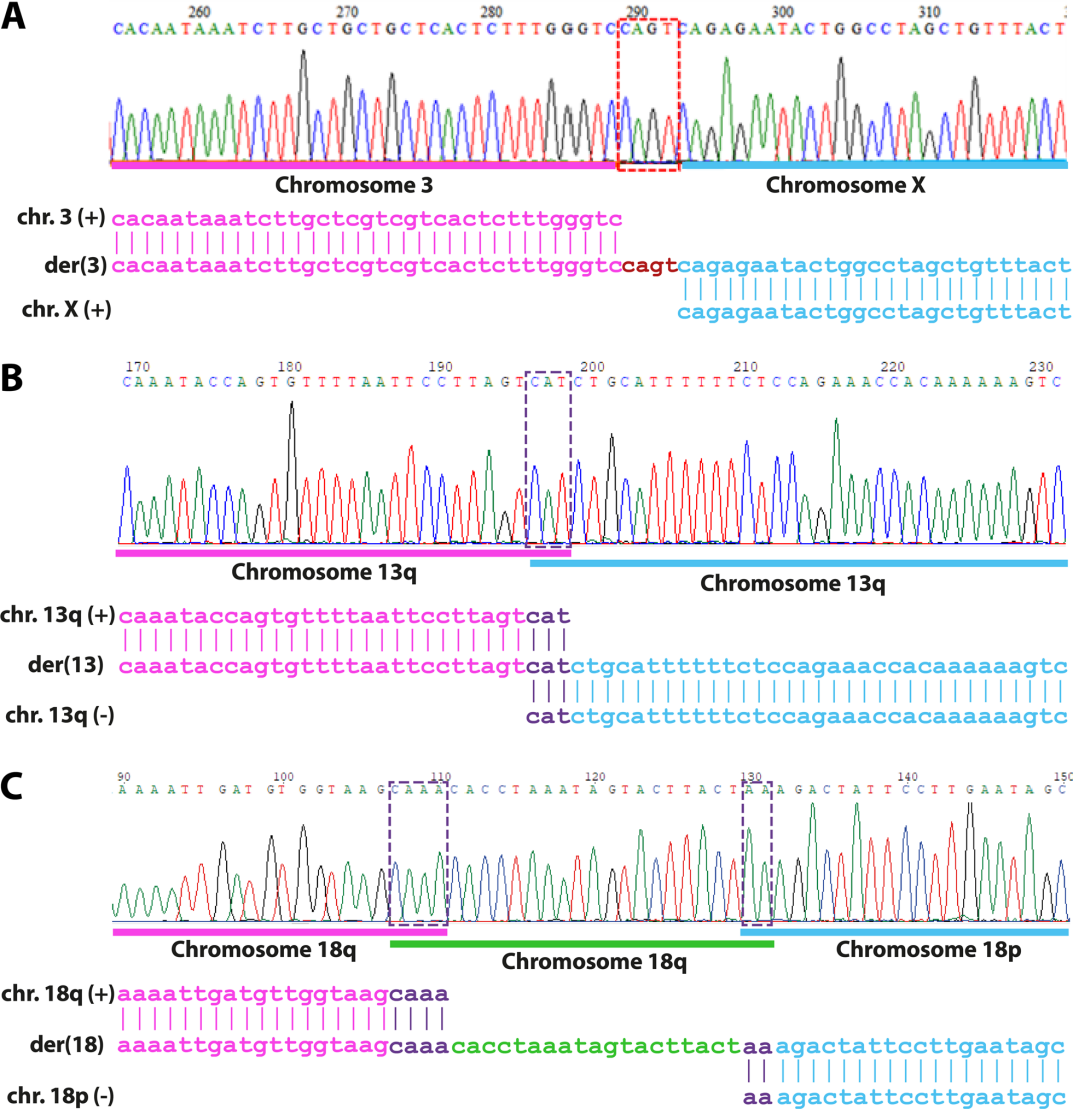
Junction point sequencing highlights different mechanisms signatures. **A** Junction point from a translocation between chromosome 3 and chromosome X with insertion of nucleotides from Non-Homologous End-Joining (NHEJ) mechanism. At the top, the chromatogram displays chromosome 3 (underlined in pink) and chromosome X (underlined in blue). At the bottom, the alignment of the derivative (der) chromosome 3 (middle) with the reference sequence chromosomes 3 (pink) and X (blue). In red, four nucleotides were added for the ligation of the chromosomes by NHEJ. **B** Junction point from an inv dup del(13q) with microhomologies from the Fold-back mechanism. At the top, the chromatogram displays the spacer (underlined in pink) and the inverted duplication (underlined in blue). At the bottom, the alignment of the derivative chromosome 13 with the reference sequence of the spacer (pink) and the inverted duplication (blue). In purple, three nucleotides of the microhomology that prompted the Fold-back mechanism's occurrence. **C** Junction point from a complex rearrangement involving both arms of chromosome 18 with an insertion from a replication (FoSTeS/MMBIR) mechanism. At the top, the chromatogram displays the normal region of the long arm (underlined in pink), the inserted region of the long arm (underlined in green), and the inverted duplication of the short arm (underlined in blue). At the bottom, the alignment of the altered derivative 18 with the reference sequence of the long arm (pink), the insertion of the long arm (green), and the short arm (blue). In purple, four nucleotides of microhomology between the two regions of the long arm, and the two nucleotides of microhomology between the insertion and the short arm

A comprehensive analysis of breakpoints and junctions is extremely necessary in order to define with more certainty the mechanism of formation of the rearrangement. High-resolution sequencing studies identify more complex breakpoints in rearrangements previously predicted as simple [7, 26]. For example, for rearrangements previously classified to be formed through NHEJ, reanalysis has revealed a new formation mechanism through MMBIR [7]. The combination of cytogenetic and next generation sequencing technologies allows for the detection and definition of the mechanisms of formation of complex chromosomal rearrangements [13]. Sequencing of the breakpoint at the nucleotide level enables the analysis of the presence of information scars from NHEJ, microhomology from MMEJ, FoSTeS, and MMBIR, and inserted segments from the replication-based mechanisms, as exemplified in Fig. 9.

The study of inv dup del rearrangements is an excellent example of the importance of using high-resolution techniques in the analysis. It is commonly reported that the U-type exchange mechanism is the most common since many studies use low-resolution techniques that miss the presence of the disomic spacer [71]. Therefore, sequencing the breakpoints is vital in order to have a thorough evaluation since the detection of spacers can help identify the actual mechanism responsible for the formation of inv dup dels [70, 71], and the actual genetic imbalance. Additionally, using techniques that can identify submicroscopic terminal deletions in ring chromosomes is strategic since their presence or absence can help differentiate the mechanism for their formation. Moreover, it is interesting to note that the discovery of chromoanagenesis was possible due to the combined use of next generation sequencing and bioinformatics tools [62].

It is critical to highlight that, even after performing Sanger sequencing of the breakpoints, it is not always possible to infer the precise mechanism of formation of chromosomal rearrangements. For example, as previously mentioned, MMBIR and FoSTeS both present microhomology at the breakpoint and therefore cannot be differentiated solely by analyzing the breakpoint sequencing result. This analysis, however, can help in identifying the type of mechanism involved, which assists in revealing how cells respond to DNA damage and repair broken ends.

## Conclusions

Several mechanisms have been proposed to explain the formation of chromosomal rearrangements. A clear criterion for the definition of such mechanisms is still lacking. This review gathers information to describe the most important mechanisms and how they impact the genome and lead to the formation of simple and complex rearrangements. We highlight the importance of performing high-resolution techniques when analyzing rearrangements to reveal the DNA features needed to propose the mechanisms involved in their formation. This way, we can better understand which mechanisms are involved in different rearrangement types, clarifying how cells deal with DNA damage in many different scenarios.

## Data Availability

All data generated or analyzed during this study are included in this published article. Further inquiries can be directed to the corresponding author.

## Abbreviations

alt-EJ or a-NHEJ: Alternative form of end-joining
BIR: Break induced replication
c-NHEJ: Canonical NHEJ
CNVs: Copy number variations
DSB: Double-strand break
FISH: Fluorescent in situ hybridization
FoSTeS: Fork stalling and template switching
HR: Homologous recombination
LCRs: Low copy repeats
LINE-1: Long interspersed element-1
MMBIR: Microhomology-mediated break-induced replication
MMEJ: Microhomology-mediated end joining
NAHR: Non-allelic homologous recombination
NGS: Next generation sequencing
NHEJ: Nonhomologous end-joining
REPD: REPeat distal
REPP: REPeat proximal
SINE: Short interspersed element
